# Direct comparisons of efficacy and safety between actinomycin-D and methotrexate in women with low-risk gestational trophoblastic neoplasia: a meta-analysis of randomized and high-quality non-randomized studies

**DOI:** 10.1186/s12885-021-08849-7

**Published:** 2021-10-18

**Authors:** Jiatao Hao, Weihua Zhou, Mengzhao Zhang, Hui Yu, Taohong Zhang, Ruifang An, Yan Xue

**Affiliations:** 1 Department of Obstetrics and Gynecology, The First Affiliated Hospital of Xi’an Jiaotong University, 277 West Yanta Road, Xi’an, Shaanxi China; 2 Department of Urology, The First Affiliated Hospital of Xi’an Jiaotong University, Xi’an, China

**Keywords:** Actinomycin-D, Gestational trophoblastic neoplasia, Low risk, meta-analysis, Methotrexate

## Abstract

**Background:**

Actinomycin-D (Act-D) and Methotrexate (MTX) are both effective first-line agents for low-risk gestational trophoblastic neoplasia (LRGTN) with no consensus regarding which is more effective or less toxic. The primary objective of this meta-analysis is to compare Act-D with MTX in the treatment of LRGTN.

**Methods:**

We systematically searched electronic databases, conferences abstracts and trial registries for randomized controlled trials (RCTs) and high-quality non-randamized controlled trials (non-RCTs), comparing Act-D with MTX for patients with LRGTN. Studies were full-text screened for quality assessment and data extraction. Eligible studies must have reported complete remission rate. A fixed-effects meta-analysis was conducted to quantify the efficacy and safety of Act-D and MTX on odds ratios (ORs) and 95% confidence intervals (95%CIs), respectively.

**Results:**

A total of 8 RCTs and 9 non-RCTs (1674 patients) were included. In terms of efficacy, Act-D is superior to MTX in complete remission (80.2% [551/687] vs 65.1% [643/987]; OR 2.15, 95%CI 1.70 to 2.73). In the stratified analysis, patients from RCTs and non-RCTs both had a better complete remission from Act-D-based regimen (RCTs: 81.2% [259/319] vs 66.1% [199/301], OR 2.17, 95%CI 1.49 to 3.16; non-RCTs: 79.3% [292/368] vs 65.0% [444/686], OR 2.14, 95%CI 1.57 to 2.92). In terms of safety, patients receiving Act-D had higher risks of suffering nausea (OR 2.35, 95%CI 1.68 to 3.27), vomiting (OR 2.40, 95%CI 1.63 to 3.54), and alopecia (OR 2.76, 95%CI 1.60 to 4.75). Notably, liver toxicity (OR 0.38, 95%CI 0.19 to 0.76) was the only one that was conformed to have a higher risk for patients receiving MTX. In addition, the pooled results showed no significant difference of anaemia, leucocytopenia, neutropenia, thrombocytopnia, constipation, diarrhea, anorexia, and fatigue between Act-D and MTX.

**Conclusions:**

Our meta-analysis suggests that Act-D had better efficacy profile in general, and MTX had less toxicities in LRGTN. Future clinical trials should be better orchestrated to provide more valid data on efficacy and toxicity.

**Supplementary Information:**

The online version contains supplementary material available at 10.1186/s12885-021-08849-7.

## Background

Gestational trophoblastic neoplasia (GTN) is a spectrum of interrelated but distinct conditions including invasive mole, choriocarcinoma, and the rare placental-site and epithelioid trophoblastic tumor, with metastatic and fatal potentiality [1]. According to a combined anatomic staging and modified World Health Organization (WHO) risk-factor scoring system that adopted by the International Federation of Gynecology and Obstetrics (FIGO) in 2002, GTN with non-metastatic (stage I) and low-risk metastatic (stages II and III, score < 7) are defined as low-risk GTN (LRGTN) [2, 3]. Over several decades, chemotherapy has already become the pivotal therapeutic strategy for LRGTN when fertility preservation is desired, with high cure rates estimated to be 80–100% even in the presence of distant metastasis, although surgical intervention may be required for complications [3–5].

Worldwide, actinomycin-d (Act-D) and methotrexate (MTX) have long been the first-line agents for LRGTN, which were first reported to be successful in the treatment of GTN around 1960s [6–8]. Up to now, several different dosing/cycling regimens for Act-D and MTX have been studied; however, the efficacy and safety of both the drug and the regimen is highly inconsistent. In 2016, a Cochrane pairwise meta-analysis by Lawrie et al. included 7 studies (577 patients) that compared MTX with Act-D, indicating that Act-D is more likely to be associated with a higher first-line complete remission rate than MTX, irrespective of the dosing and cycling. Low-certainty evidence suggested that there was no significant difference in adverse events between Act-D and MTX; however, the five-day Act-D regimen (5d-IV Act-D) may cause more mucositis and alopecia than eight-day MTX-folinic acid regimen (MTX-FA) when dosages and cycles were considered [9]. To further conduct comparisons of these different regimens, Li et al. performed a network meta-analysis of 7 randomized controlled trials (RCTs) and 4 retrospective studies to compare all single-agent Act-D-based and MTX-based regimens, and found that Act-D-based regimens (5d-IV Act-D and IV Act-D) were more effective than MTX-based regimens. In contrast, patients treated with MTX-based regimens had higher probability of suffering gastrointestinal toxicities such as nausea and vomiting [10]. Although antitumor advantages can be seen from Act-D according to published meta-analyses, we still cannot draw conclusions of who is safer.

Taken together, previous meta-analyses on efficacy and safety mainly focused on specific regimens, failing to cover all regimens. Therefore, a complete picture of efficacy and toxicity related to Act-D and MTX from RCTs and high-quality non-randomized studies (non-RCTs) is warranted. Here, we perform a comprehensive meta-analysis of 17 studies comparing the efficacy and safety of Act-D and MTX, with the aim of providing overall efficacy and safety profiles and aiding decision-making for patients, clinicians and reference centers.

## Methods

The present study was performed in line with the PRISMA (preferred reporting items for systematic reviews and meta-analysis) [11].

### Data searches and information sources

**Fig. 1:**
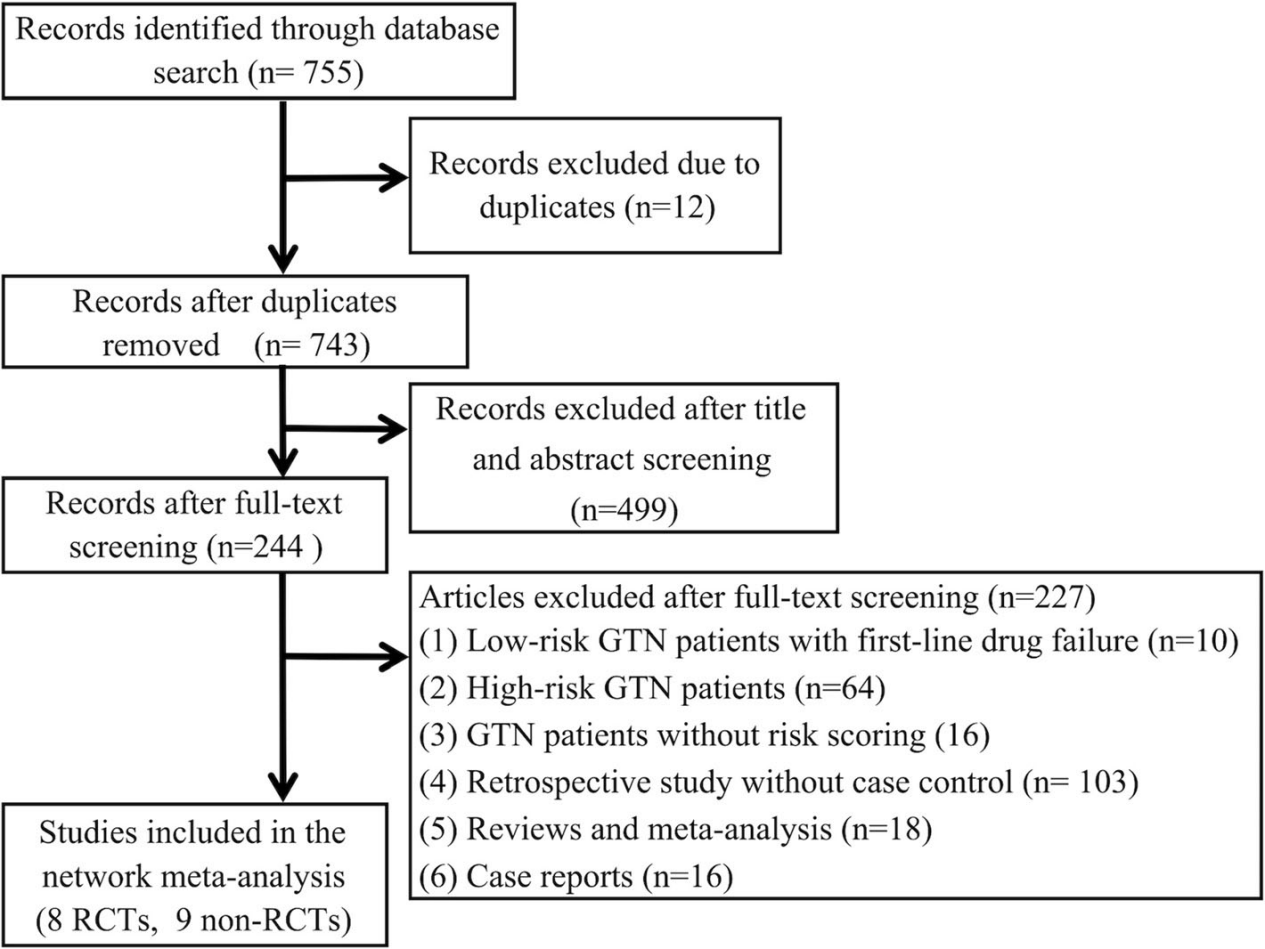
The flow diagram of the selection process for studies included in the present meta-analysis

Two investigators independently searched the medical databases including PubMed/Medline, Embase, Cochrane Library and Web of Science for candidate articles published in English from inception to August 2020, using the following prespecified search terms and their combinations: ‘gestational trophoblastic disease’, ‘gestational trophoblastic neoplasia’, ‘gestational trophoblastic tumor’, ‘gestational trophoblastic neoplasm’, ‘invasive mole’, ‘choriocarcinoma’, ‘low risk’, ‘actinomycin-D’, ‘Act-D’, and ‘methotrexate’, and ‘MTX’. After computerized searching, the clinical trial registries (www.clinical-trials.gov), conference proceedings, reviews and meta-analyses were also examined for potentially relevant publications that omitted in initial literature retrieval. The reviewers then assessed the full text and relevant articles cited as references to include any that met criteria for eligibility in the quantitative synthesis. Figure 1 presents the study selection flowchart.

### Eligibility criteria for study selection

Inclusion criteria were prespecified according to the published meta-analyses [9, 10]. Eligible studies were RCTs and non-RCTs comparing an Act-D-based regimen directly to a MTX-based regimen for first-line treatment of LRGTN patients which were defined based on the FIGO/WHO 2000 scoring system or other scoring criteria that proven to be reliable. For clinical outcomes, proportion of individuals who developed complete remission should be reported to assess the efficacy of drugs. The included studies should provide information about the characteristics of patients and if they were matched for potentially confounding variables in each treatment group. To identify studies from the same cohort or institution, only the most recent or the most informative publication was included. We did not exclude retrospective studies because of the exiguity of RCTs of GTN. We excluded conference abstracts, posters, and presentations of ongoing RCTs because these brief reports did not contain detailed data.

### Data extraction and definitions

Two authors independently evaluated the main text and supplementary materials to extract detailed data on the first author, year of publication, country of origin, study design, total number of patients, number of patients in efficacy and safety analysis, arms and chemotherapy regimens, and the frequency of complete remission and specific adverse events. Complete remission rate was selected as the primary outcome and based on the number of patients who reached complete remission and the total number of patients who received treatment. We used odds ratios (ORs) and 95% confidence intervals (95%CIs) as summary statistics to quantify the efficacy and toxicity in this meta-analysis. We selected toxicities that were evaluated using the Common Terminology Criteria for Adverse Events (CTCAE), WHO, and Gynecologic Oncology Group (GOG) toxicity criteria. In addition, few studies that did not mention the methods for collecting adverse events, possibly depending on investigators’ evaluation or self-reporting by patients, were also included. Regardless of the adverse event grading, general safety was used to indicate the overview of the toxicities without distinguishing between their specific classifications. Proportion of patients with specific toxicities was used to quantity the safety of the agents.

### Quality assessment

We assessed the risk of bias for individual RCTs based on the original study and supplementary materials by adopting the Cochrane Risk of Bias Tool which includes the following domains: random sequence generation, allocation concealment, blinding method, assessment of outcomes, and reporting of results. Each item was associated with the risk of bias classified as yes, no or unclear [12]. The modified Jadad scale was implemented to score the method quality of included RCTs. Four points and over of the modified Jadad score indicate high quality of method, and three points and under mean low quality [13]. Unfortunately, there is no universal method to evaluate quality in non-RCTs. For the purposes of assessment of risk bias in non-RCT studies, we decided to use the modified methodological index for non-randomised studies (modified MINORS), which was adopted in the published study [14, 15]. We did not excluded studies based on a Jadad score and the modified MINORS for overall quality. All discrepancies in data searches, study selection, data extraction, and quality assessment were resolved by consensus and in consultation with a third author (An).

### Data synthesis

In this meta-analysis, ORs and 95%CIs were used as summary statistics to quantify the efficacy and toxicity of Act-D and MTX. ORs greater than one represented a treatment benefit favoring Act-D and a safety profile disfavoring Act-D. We generated the pooled ORs and 95%CIs of complete remission and all-grade adverse events. RCTs and non-RCTs were first analyzed conjunctively using subgroup analyses, and then separated using a fixed-effects model. This allowed us to see the contrasts between the results of RCTs with those of non-RCTs, and made possible data combination of RCTs and non-RCTs to obtain pooled estimates. We first used the fixed-effects model to merge the data, otherwise the random-effects model was applied in case of significant between-trial heterogeneity variances which were quantified using the I^2^ inconsistency test [16]. The heterogeneity was regarded as substantial if the I^2^ was greater than 50%. Forest plots were constructed to provide graphical presentations for all meta-analyses. In addition, we conducted sensitivity analyses to assess the stability of results by using the leave-one-out method wherein the line in horizontal box plot indicated the result for all studies. The publication bias was assessed using Begg’s adjusted rank correlation test (Z-statistics) [17] and quantified by Egger’s linear regression test (t-statistics) [18], and illustrated using the funnel plots. Stata version 11.0 (Stata Corporation, College Station, TX, USA) was used to perform all the meta-regression and subgroup analysis of ORs. All statistical tests were two-sided and the *P* value threshold for statistical significance was set at 0.05 for effect sizes.

## Results

### Studies selection and characteristics

After full-text screening, we ultimately included 17 studies that met the eligibility criteria in the present analysis: 8 RCTs [6, 19–25] and 9 non-RCTs [26–34]. The flow diagram in Fig. 1 details the selection process. Table 1 summarizes main features regarding all included studies. Given the context that GTN is rare and reference centers have preferred chemotherapy protocols, only one study was a multi-nation trial amongst the 8 RCTs. In addition, 11 studies were done in Asia countries, 3 came from Brazil, and 2 were from U. S and Netherlands, respectively. Differing from the published network meta-analysis, the definition of LRGTN in the included studies was based on either FIGO/WHO 2000 scoring system or the Hammond criteria. For the meta-analysis of first-line and single-agent regimens, studies comparing the use of Act-D-based regimen (5d-IV Act-D and pulsed IV Act-D) with MTX-based regimen (5d-IV MTX, 5d-IM MTX, w-IM MTX, and MTX-FA) were included. 15 studies contained one group of comparison: MTX or MTX/FA vs Act-D. Two non-RCTs reported three drug groups including MTX, MTX/FA, and Act-D. We divided the three drugs into two groups of comparisons in the analysis: MTX vs Act-D and MTX/FA vs Act-D. A total of 1674 patients were included in the meta-analysis. Eight RCTs contributed 620 cases, 319 and 301 of them allocated in the Act-D group and MTX group, respectively. In the analysis of non-RCTs, 1054 patients were included, 368 received Act-D-based treatment, whereas 686 patients received MTX-based treatment.

**Table 1:** Characteristics of the studies included in the meta-analysis

Author (year)	Region	Type	Actinomycin D-based regimen			Methotrexate-based regimen		
			Treatment	No.	CR	Treatment	No.	CR
Kang (2019)	China	RCT	Act-D, IV, 10 μg/kg daily for 5 days	49	43	MTX, IM, 0.4 mg/kg daily for 5 days	49	41
Yarandi (2016)	Iran	RCT	Act-D, IV, 1.25 mg/m2 biweeklyAct-D, IV, 1.25 mg/m2 biweekly	30	24	MTX, IV, 0.4 mg/kg daily for 5 days	32	25
Shahbazian (2014)	Iran	RCT	Act-D, IV, 1.25 mg/m2 biweeklyAct-D, IV, 1.25 mg/m2 biweekly	15	13	MTX, IM, 40 mg/m2 weeklyMTX, IM, 40 mg/m2 weekly	15	8
Mousavi (2012)	Iran	RCT	Act-D, IV, 1.25 mg/m2 biweeklyAct-D, IV, 1.25 mg/m2 biweekly	50	45	MTX, IM, 0.4 mg/kg daily for 5 days	25	17
Lertkhachonsuk (2009)	Thailand	RCT	Act-D, IV, 10 μg/kg daily for 5 days	20	20	MTX, IM, 1 mg/kg on days 1, 3, 5 and 7 + FA, IM, 0.1 mg/kg on days 2, 4, 6 and 8	19	14
Osborne (2011)	Multi-nation	RCT	Act-D, IV, 1.25 mg/m2 biweeklyAct-D, IV, 1.25 mg/m2 biweekly	109	76	MTX, IM, 30 mg/m2 weeklyMTX, IM, 30 mg/m2 weekly	107	57
Gilani (2005)	Iran	RCT	Act-D, IV, 1.25 mg/m2 biweeklyAct-D, IV, 1.25 mg/m2 biweekly	18	16	MTX, IM, 30 mg/m2 weeklyMTX, IM, 30 mg/m2 weekly	28	14
Schink (2020)	U.S.	RCT	Act-D, IV, 1.25 mg/m2 biweeklyAct-D, IV, 1.25 mg/m2 biweekly	28	22	MTX, IV, 0.4 mg/kg daily for 5 days	26	23
Verhoef (2017)	Netherlands	Non-RCT	Act-D, IV, 1.25–2 mg/m2 biweeklyAct-D, IV, 1.25–2 mg/m2 biweekly	34	29	MTX, IM, 50 mg biweekly	4	1
Al-Husaini (2014)	Arabia	Non-RCT	Act-D, IV, 1.25 mg/m2 biweekly or Act-D, IV, 0.5 mg daily for 5 daysAct-D, IV, 1.25 mg/m2 biweekly or Act-D, IV, 0.5 mg daily for 5 days	23	20	MTX, IM, 1 mg/kg weekly or MTX, IM, 1 mg/kg on days 1, 3, 5 and 7	73	39
Uberti (2015)	Brazil	Non-RCT	Act-D, IV, 1.25–2 mg/m2 biweekly	79	53	MTX, IM, 1 mg/kg on days 1, 3, 5 and 7 + FA, Oral, 15 mg on days 2, 4, 6 and 8	115	87
Abrao (2008)	Brazil	Non-RCT	Act-D, IV, 12 μg/kg daily for 5 days	42	30	MTX, IM, 20 mg/m2 daily for 5 daysMTX, IM, 20 mg/m2 daily for 5 days	42	29
Yarandi (2008)	Iran	Non-RCT	Act-D, IV, 1.25 mg/m2 biweeklyAct-D, IV, 1.25 mg/m2 biweekly	50	45	MTX, IM, 30 mg/m2 weeklyMTX, IM, 30 mg/m2 weekly	81	39
Baptista (2012)	Brazil	Non-RCT	Act-D, IV, 12 μg/kg daily for 5 days	20	18	MTX, IM, 50 mg + FA, oral, 15 mg, for 8 days	20	10
Lee (2017)	South Korea	Non-RCT	Act-D, IV, 1.25 mg/m2 biweekly or Act-D, IV, 12 μg/kg daily for 5 days	18	15	MTX, IM, 1 mg/kg on days 1, 3, 5 and 7 + FA, IM, 0.1 mg/kg on days 2, 4, 6 and 8 or MTX, IM, 50 mg/m2 weekly	53	33
Matsui (2005)	Japan	Non-RCT	Act-D, IV, 8.5-10μg/kg daily for 5 days	26	20	MTX, IM, 0.35–0.4 mg/kg daily for 5 days	133	91
Matsui (2005)	Japan	Non-RCT	Act-D, IV, 8.5-10μg/kg daily for 5 days	26	20	MTX, IM, 0.85-1 mg/kg on days 1, 3, 5 and 7 + FA, IM, 85-100μg/kg on days 2, 4, 6 and 8	24	14
Matsui (1998)	Japan	Non-RCT	Act-D, IV, 8.5-10μg/kg daily for 5 days	25	21	MTX, IM, 0.35–0.4 mg/kg daily for 5 days	121	89
Matsui (1998)	Japan	Non-RCT	Act-D, IV, 8.5-10μg/kg daily for 5 days	25	21	MTX, IM, 0.85-1 mg/kg on days 1, 3, 5 and 7 + FA, IM, 85-100μg/kg on days 2, 4, 6 and 8	20	12

*CR* Complete remission, *RCT* Randomized controlled trials, *non-RCT* non-randomized studies, *Act-D* Actinomycin-d, *MTX* Methotrexate, *IM* Intramuscular, *IV* Intravenous

### Assessment of risk of bias

Table 2 depicts the results of quality assessment. Modified Jadad scale indicated that 8 RCTs were high quality with scores ranging from 4 to 7. Most RCTs suffered from methodologic weaknesses frequently seen in allocation concealment and blinding method domains. The treating physicians or the patients in most studies were not blinded to the allocated regimens because of inherent complexity of blinding between groups. The treatment assignments were also not concealed from institutions because of preferred regimens. Only one RCT did not mention the randomization of intervention [21]. All RCTs identified key outcomes that have been reported for first-line, single-agent chemotherapy for LRGTN patients, and were free of selective reporting. Table 3 presents the assessment of all non-RCTs. The modified MINORS score of the included non-RCTs ranged from 11 to 16. In general, they are all retrospective studies with nature drawbacks in prospective data collection, with the exception of one prospective study that could yield data prospectively. The detailed features of these two quality assessment tools can be accessed through the published articles.

**Table 2:** Quality assessment of the 8 randoized controlled trials for the meta-analysis

Study	Adequate random sequence generation	Allocation concealment	Blinding method	Adequate assessment of each outcome	Free of selective reporting	Modified Jadad score
Kang (2019)	Y	U	U	Y	Y	5
Yarandi (2016)	Y	Y	Y	Y	Y	7
Shahbazian (2014)	U	U	U	Y	Y	4
Mousavi (2012)	Y	U	U	Y	Y	5
Lertkhachonsuk (2009)	Y	U	U	Y	Y	5
Osborne (2011)	Y	N	Y	Y	Y	5
Gilani (2005)	Y	U	U	Y	Y	5
Schink (2020)	Y	U	U	Y	Y	5

*U* Unclear, *Y* Yes, *N* No

**Table 3:** Modified MINORS scores of all eligible non-randomised comparative studies in this meta-analysis

Study	Consecutive patients	Prospective data collection	Reported end-points	Unbiased outcome evaluation	Appropriate controls	Contemporary groups	Groups equivalent	Sample size	Score
Verhoef (2017)	2	0	2	1	2	2	1	1	11
Al-Husaini (2014)	2	0	2	1	2	2	1	1	11
Uberti (2015)	2	0	2	1	2	2	2	1	12
Abrao (2008)	2	0	2	2	2	2	2	2	14
Yarandi (2008)	2	0	2	2	2	2	2	2	14
Baptista (2012)	2	2	2	2	2	2	2	2	16
Lee (2017)	2	0	2	1	2	2	1	1	11
Matsui (2005)	2	0	2	1	2	2	1	1	11
Matsui (1998)	2	0	2	1	2	2	1	1	11

### Meta-analysis for efficacy profile

**Fig. 2:**
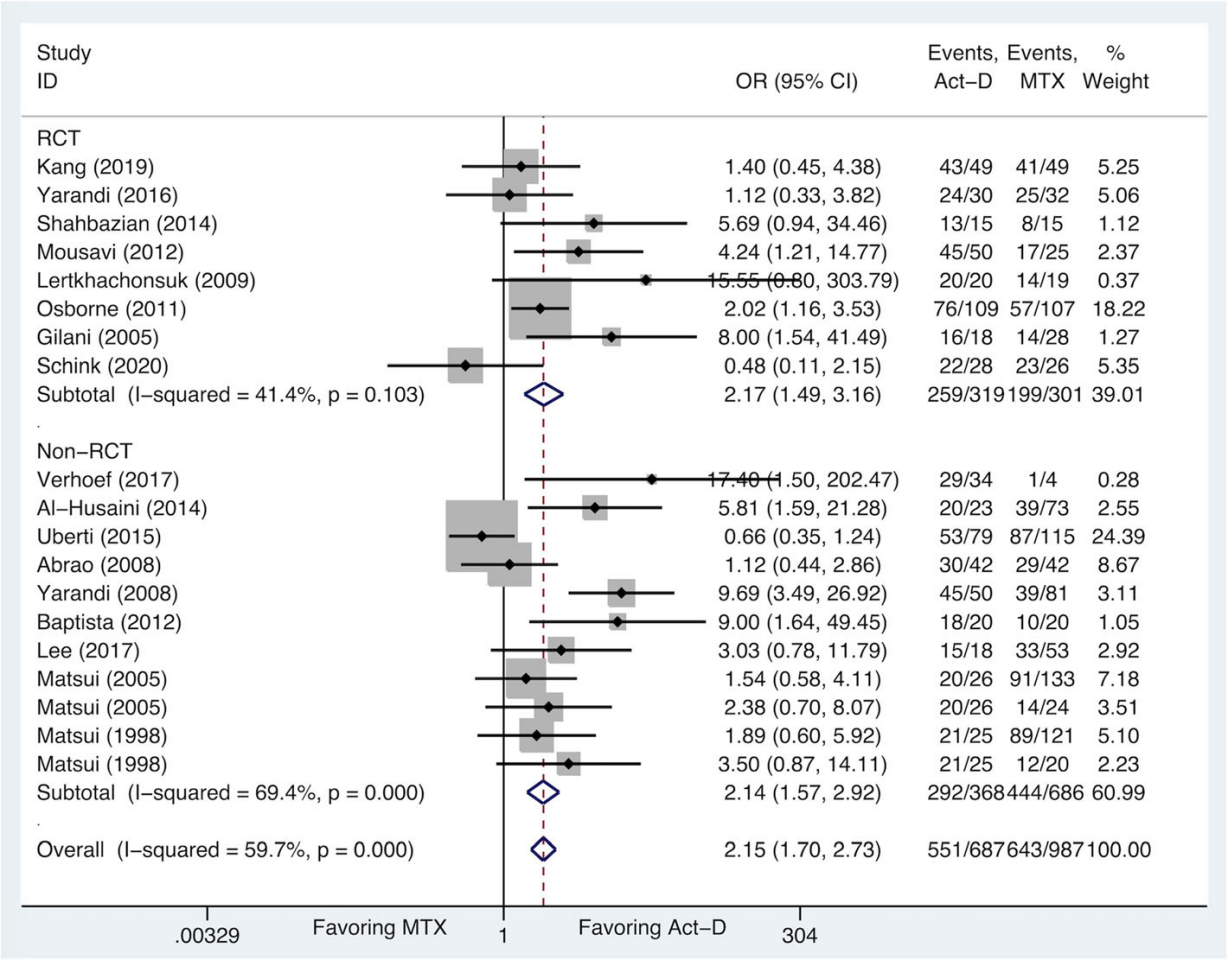
Comparisons of ORs according to drugs and study type (fixed-effects model)

The upfront drug-based meta-analysis was conducted to compare the proportion of complete responders to Act-D-based regimen and MTX-based regimen. The overall analysis demonstrated that Act-D-based regimen is superior to MTX-based regimen in complete remission (80.2% [551/687] vs 65.1% [643/987]; OR 2.15, 95%CI 1.70 to 2.73), although there was substantial variation between the results of the individual studies (I^2^ = 59.7%, *P* = 0.000). When the random-effects model was applied, the superiority of complete remission seen for Act-D-based regimen remained (OR 2.51, 95%CI 1.63 to 3.86). In the stratified analysis, we grouped studies with RCTs and non-RCTs separately. In RCTs, Act-D-based regimen showed a significant advantage in complete remission (81.2% [259/319] vs 66.1% [199/301]; OR 2.17, 95%CI 1.49 to 3.16) with no evidence of heterogeneity (I^2^ = 41.4%, *P* = 0.103). For patients in non-RCTs, there was also a better complete remission from Act-D-based regimen (79.3% [292/368] vs 65.0% [444/686]; OR 2.14, 95%CI 1.57 to 2.92). Although the relationship was inconsistent across studies (I^2^ = 69.4%, *P* = 0.000), the results did not change significantly when the random-effects model was applied (OR 2.77, 95%CI 1.47 to 5.21) (Fig. 2 and Fig. S1).

### Meta-analysis for hematological toxicities

**Fig. 3:**
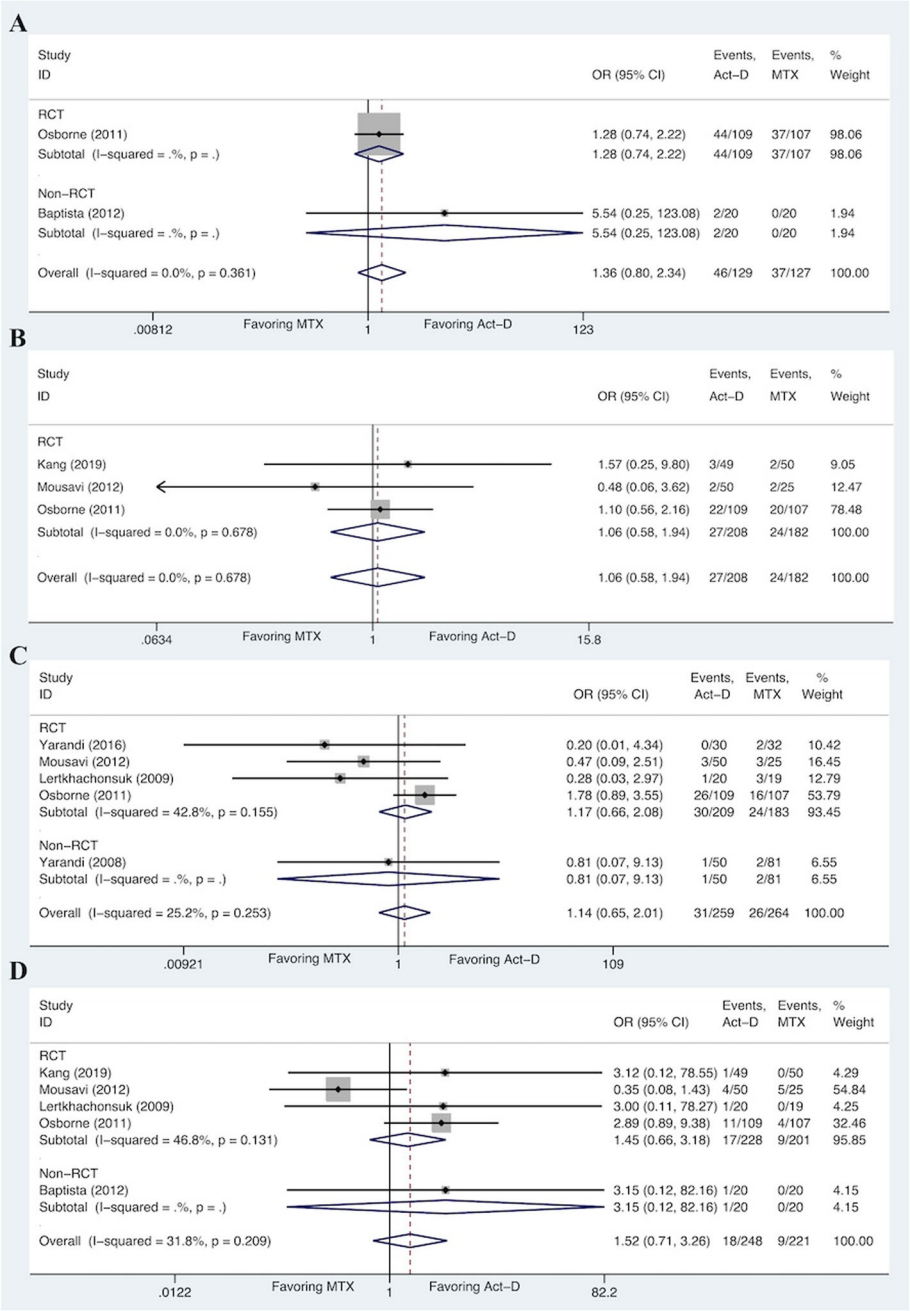
Forest plots of pooled ORs for anaemia (**A**), leucopenia (**B**), neutropenia (**C**), and thrombocytopenia (**D**) (fixed-effects model)

**Fig. 4:**
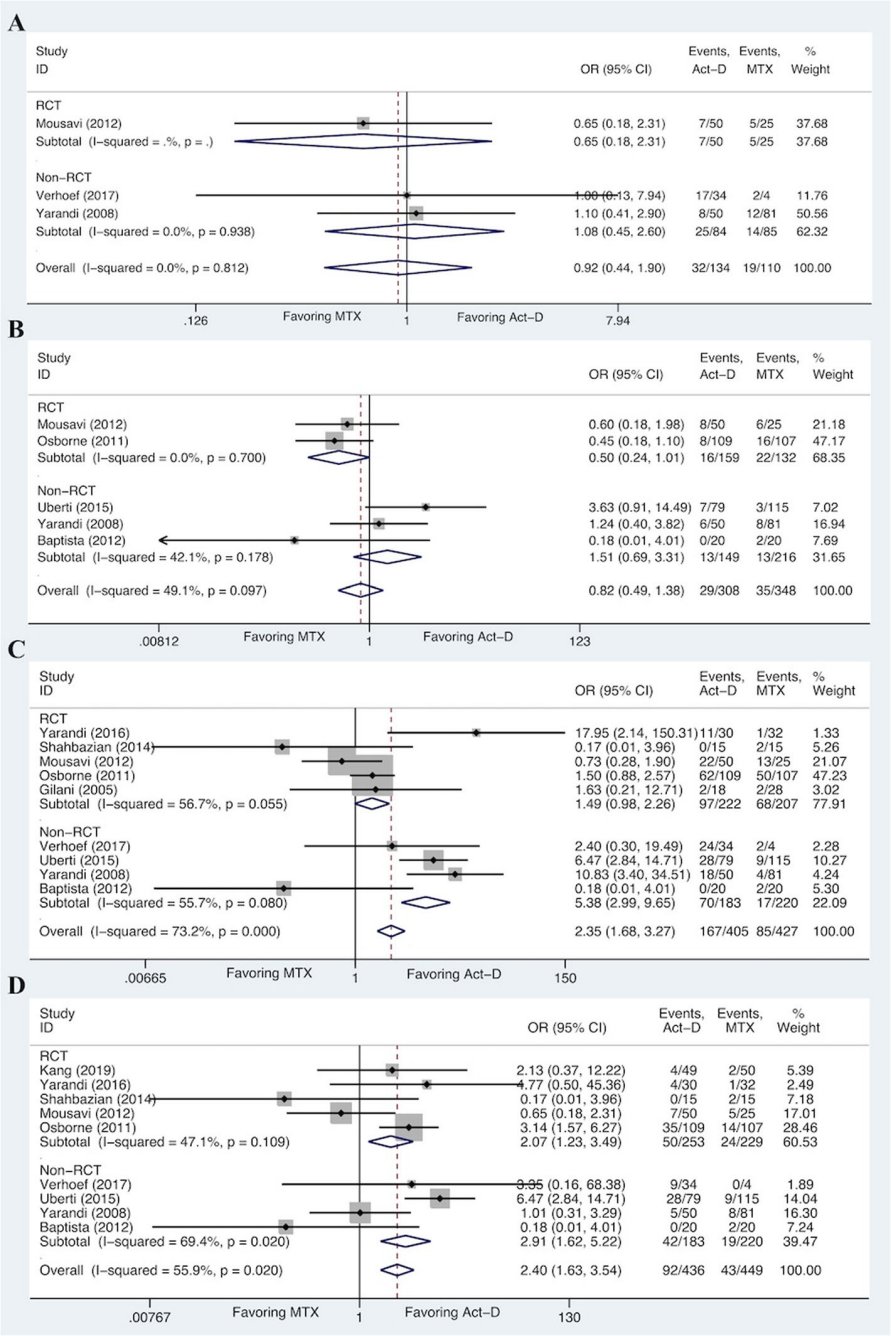
Forest plots of pooled ORs for constipation (**A**), diarrhea (**B**), nausea (**C**), and vomiting (**D**) (fixed-effects model)

**Fig. 5:**
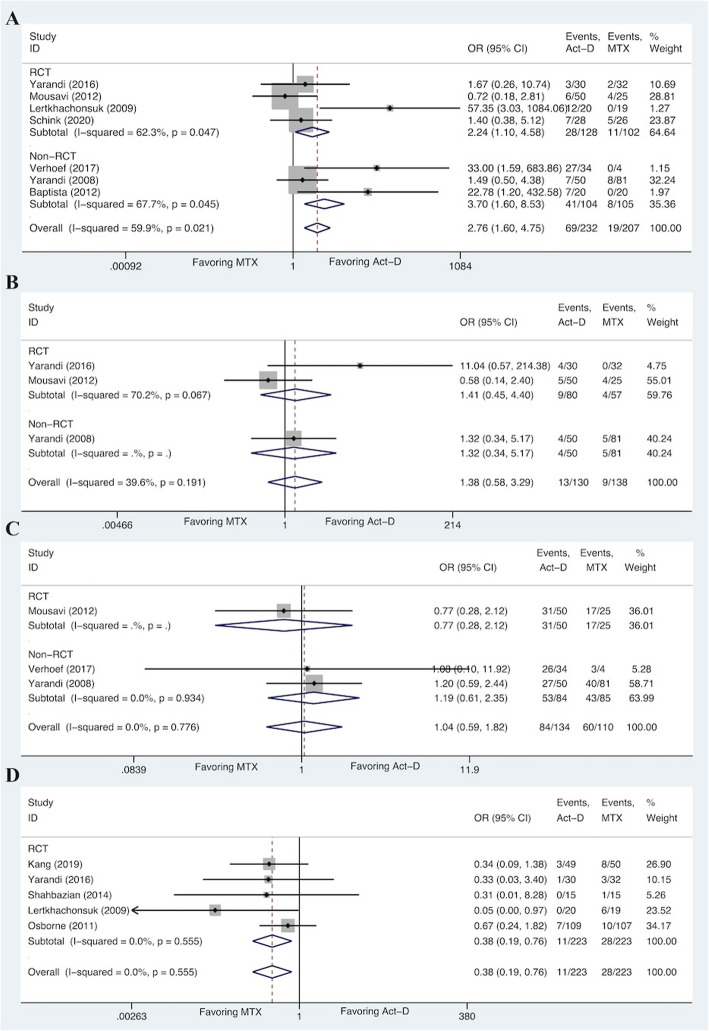
Forest plots of pooled ORs for alopecia (**A**), anorexia (**B**), fatigue (**C**), and liver toxicity (**D**) (fixed-effects model)

Figure 3 detailed the hematological toxicities of Act-D-based regimen and MTX-based regimen. The overall analyses did not show the significant difference of anaemia (OR 1.36, 95%CI 0.80 to 2.34; I^2^ = 0.0%, *P* = 0.361), leucocytopenia (OR 1.06, 95%CI 0.58 to 1.94; I^2^ = 0.0%, *P* = 0.678), neutropenia (OR 1.14, 95%CI 0.65 to 2.01; I^2^ = 25.2%, *P* = 0.253), and thrombocytopnia (OR 1.52, 95%CI 0.71 to 3.26; I^2^ = 31.8%, *P* = 0.209) for each one of the groups with no significant between-study heterogeneity. From the supplementary materials (Table 4), the pooled incidence of anaemia (35.7% vs 29.1%), neutropenia (12.0% vs 9.8%), and thrombocytopnia (7.3% vs 4.1%) for Act-D-based regimen was higher than those for MTX-based regimen, whereas the incidence of leucocytopenia (13.2% vs 13.0%) for MTX-based regimen was slightly higher than that for Act-D-based regimen; however, these results were not reported in the previous network meta-analyses for LRGTN [9, 10]. Further details of subgroup analyses for all toxicities are available in Figs. 3, 4 and 5 and supplementary materials (Fig. S2 and S3).

**Table 4:** Pooled incidences of selected toxicities

Adverse events	Studies(t)	Act-D-based regimen (***n*** = 687)		MTX-based regimen (***n*** = 987)	
		Patients(n_**1**_/n_**2**_)	%	Patients(n_**1**_/n_**2**_)	%
**Hematologic disorders**					
Anemia	2	46/129	35.7	37/127	29.1
Leucocytopenia	3	27/208	13.0	24/182	13.2
Neutropenia	5	31/259	12.0	26/264	9.8
Thrombocytopnia	5	18/248	7.3	9/221	4.1
**Gastrointestinal disorders**					
Constipation	3	32/134	23.9	19/110	17.3
Diarrhea	5	29/308	9.4	35/348	10.1
Nausea	9	167/405	41.2	85/427	19.9
Vomiting	9	92/436	21.1	43/449	9.6
**Others**					
Alopecia	7	69/232	29.7	19/207	9.2
Anorexia	3	13/130	10.0	9/138	6.5
Fatigue	3	84/134	62.7	60/110	54.5
Liver toxicity	5	11/223	4.9	28/223	12.6

*Act-D* Actinomycin D, *MTX* Methotrexate, *t* The number of studies reporting the toxicity, *n* Total number of enrolled patients, *n1* The number of patients with adverse events, *n2* the total number of patients from studies reporting the toxicity

### Meta-analysis for gastrointestinal toxicities

The overall analyses of gastrointestinal toxicities are shown in Fig. 4. The pooled results demonstrated that there was no significant difference of constipation (OR 0.92, 95%CI 0.44 to 1.90; I^2^ = 0.0%, *P* = 0.812) and diarrhea (OR 0.82, 95%CI 0.49 to 1.38; I^2^ = 49.1%, *P* = 0.097) between Act-D-based regimen and MTX-based regimen, with no evidence of between-study heterogeneity. The pooled incidence of constipation (23.9% vs 17.3%) for Act-D-based regimen was higher than that of MTX-based regimen; however, MTX-based regimen had a higher incidence of diarrhea (10.1% vs 9.4%) than Act-D-based regimen (Table 4). For patients receiving Act-D-based regimen, there were significant higher risks of suffering nausea (OR 2.35, 95%CI 1.68 to 3.27) and vomiting (OR 2.40, 95%CI 1.63 to 3.54), whereas there was moderate evidence of heterogeneity among the studies (nausea I^2^ = 73.2%, *P* = 0.000; vomiting I^2^ = 55.9%, *P* = 0.020). When the random-effects model was applied, the pooled data changed significantly for nausea (OR 2.32, 95%CI 0.98 to 5.46) and vomiting (OR 1.85, 95%CI 0.88 to 3.89) (Fig. S2). In line with the results from fixed-effects model, pooled incidences of nausea (41.2% vs 19.9%) and vomiting (21.1% vs 9.6%) for Act-D-based regimen were higher than those for MTX-based regimen (Table 4).

### Meta-analysis for toxicities from other systems

We selected four toxicities that were categorized into other systems. A significant higher risk for the Act-D-based regimen of developing alopecia (OR 2.76, 95%CI 1.60 to 4.75) was found in the meta-analysis, although there was a moderate heterogeneity among studies (I^2^ = 59.9%, *P* = 0.021) (Fig. 5). However, the pooled result (OR 3.01, 95%CI 1.02 to 8.86) did not change significantly when the random-effects model was applied (Fig. S3). Similarly, pooled incidence of alopecia for Act-D-based regimen was 29.7%, which was higher than that of MTX-based regimen (9.2%) (Table 4). On the contrary, no significant differences appeared in anorexia (OR 1.38, 95%CI 0.58 to 3.29; I^2^ = 39.6%, *P* = 0.191) and fatigue (OR 1.04, 95%CI 0.59 to 1.82; I^2^ = 0.0%, *P* = 0.776), even if higher pooled incidences of anorexia (10.0% vs 6.5%) and fatigue (62.7% vs 54.5%) were observed in Act-D-based regimen. Notably, liver toxicity (OR 0.38, 95%CI 0.19 to 0.76; I^2^ = 0.0%, *P* = 0.555) was the only one that was conformed to have a higher risk for patients receiving MTX-based regimen, which was consistent with the pooled incidence (12.6% for MTX-based regimen vs 4.9% for Act-D-based regimen) (Fig. 5 and Table 4).

### Sensitivity analysis and publication bias

Sensitivity analyses were conducted to evaluate the stability and reliability of the pooled ORs (for complete remission and toxicities). As shown in Fig. S4, S5, S6 and S7, the horizontal box plots of leave-one-out method revealed that ORs of anaemia and anorexia were seemingly influenced by a single study. We, therefore, carried out subgroup analyses according to the study type, which showed no significant difference among the RCTs and non-RCTs for anaemia and anorexia. The pooled ORs for anaemia were similar for RCTs (OR 1.28, 95%CI 0.74 to 2.22) and non-RCTs (OR 5.54, 95%CI 0.25 to 123.08) (Fig. 3). Similar result was obtained for anorexia (RCTs: OR 1.41, 95%CI 0.45 to 4.40; non-RCTs: OR 1.32, 95%CI 0.34 to 5.17) (Fig. 5). Furthermore, we assessed the publication bias of included studies using funnel plots and Begg’s adjusted rank correlation test. For ORs, the funnel plots exhibited a symmetrical distribution, indicating the absence of publication bias, which was further confirmed with the Begg’s adjusted rank correlation test (*P* > 0.05) (Fig. S4, S5, S6, S7 and Table S1).

## Discussion

Act-D and MTX act through distinct anti-tumor mechanisms and should be compared with regards to efficacy and safety. With the paucity of comprehensive comparisons of therapeutic effectiveness and toxicity for the two drugs as first-line chemotherapy for LRGTN patients, we included 8 RCTs and 9 non-RCTs (1674 patients) in present meta-analysis. Given that LRGTN patients who like to preserve their fertility would firstly receive single-agent chemotherapies, such as Act-D and MTX, and these agents have several dosing/cycling options, our study regarded all Act-D-based and MTX-based regimens as one entity, respectively [3, 4]. Similar to observations from previous meta-analysis investigating the efficacy of Act-D and MTX [9, 10], our results confirmed that Act-D had greater superiority in terms of complete response than MTX, irrespective of dosage and cycle. Of note, we have obtained some unique findings that some toxicities such as nausea, vomiting, and alopecia are more common in LRGTN patients treated with Act-D-based regimens, and liver toxicities were more commonly associated with MTX-based regimens. However, a previous net-work meta-analysis by Li et al. found that nausea and vomiting were more frequently observed in 5d-IM MTX regimen, which was inconsistent with our results [10]. With a tailored study design in which LRGTN patients diagnosed according to the Hammond criteria were also included, our pair-wise meta-analysis included more studies and patients, and gave more comprehensive comparisons. Accordingly, the present meta-analysis will aid reference centers and patients to select more effective agents and optimize toxicity management for patients with LRGTN.

Patients with GTN are now identified as a lucky group that have preferable responses to chemotherapy. The prognosis has significantly improved over the past decades from almost hopeless to a new situation in which the majority of the GTN patients can achieve complete remission, even if a metastatic condition exists. Particularly, LRGTN patients may more likely yield better cure rates to single-agent regimens such as Act-D and MTX, with resulting survival rates approaching 100% [3–5]. Currently, Act-D and MTX have been administered in various regimens with different dosages and cycles, which have been proposed by different reference centers. Although RCTs and retrospective studies have investigated different regimens of Act-D and MTX, there is still no universal consensus on the optimal dosing and cycling for both Act-D and MTX, which was reflected in variability of complete remission rates in different studies. From an overall perspective, our study is an opportunity to move away from comparisons that have centered on specialized regimens, to focus more on the entity instead. The results of our analysis indicated that Act-D-based regimens are more effective than MTX-based regimens as first-line chemotherapy for LRGTN patients. Given the fact that GTN is a rare disease and a limited number of patients are available for randomized clinical studies [35], we included both RCTs and non-RCTs to pool the data. Impressively, stratified analysis showed that pooled OR for complete remission of RCTs was similar to that of non-RCTs, although the quality assessment indicated some methodologic defects of nature for non-RCTs. With this small effort, both randomized trials and nonrandomized or retrospective studies are warranted, and should be better orchestrated for LRGTN patients, to not only share valuable clinical experiences but also explore more possibilities of treatment.

Since the overwhelming majority of LRGTN patients have been able to attain complete remission from first-line single-agent chemotherapy and drug resistances could be successfully salvaged, the ideal drugs and regimens for LRGTN are considered to be minimizing toxicities and maximizing efficacy [34]. Of the toxicities reported in the included studies, a substantial proportion were hematological and gastrointestinal, and the remainder were a mixture that affected other organs. As for hematological toxicities, the forest plots of ORs did not demonstrate differences between Act-D and MTX, but the pooled incidences of anaemia, neutropenia, and thrombocytopenia for Act-D were slightly higher, with the exception of leucocytopenia. Myelosuppression of Act-D was not merely seen in GTN patients. One study found that 39% of the breast cancer patients who had received Act-D chemotherapy suffered mild to moderate myelosuppression [36]. However, Act-D was commonly used for GTN patients and relatively less-used for other tumors, particularly highlighting the importance of managing hematological toxicities of Act-D in GTN patients. In terms of gastrointestinal toxicities, patients treated with Act-D significantly suffered more nausea and vomiting, which was confirmed by the forest plots and pooled incidences. However, the network meta-analysis by Li et al. suggested that Act-D was less toxic than MTX, although some Act-D-based regimens seemingly lost favor because of nausea and alopecia according to previous studies [37]. In addition, alopecia and anorexia were more frequently seen in patients treated with Act-D, indicating that Act-D may affect skin and nutrition metabolism. Although the toxicities are clearly linked with additional disease and financial burden [38], some studies did not report toxicity data and there were only 12 included studies explored the toxic effects of Act-D and MTX. Therefore, comprehensive characterization of toxicities would be needed for recognizing adverse events and enhancing life quality in the future clinical studies [39].

Patients with LRGTN have the option to start a well-tolerated single-agent regimen such as MTX and Act-D which will help achieve a complete remission. With many options of cytotoxic drugs in the clinical practice, choosing drugs based on efficacy, toxicity, and cost can strive for the maximum benefit for patients. According to this meta-analysis, MTX was more commonly used as initial treatment for LRGTN patients than Act-D. However, about 10% of LRGTN patients develop chemoresistance or relapse after initial chemotherapy [40]. MTX resistance has been successfully treated with single-agent regimens using Act-D which is the most commonly recommended choice because of its reliable curative effect. However, scaling up to Act-D from MTX would lead to more toxicity profiles, particularly nausea, vomiting, and alopecia. Some other single-agent regimens such as etoposide, carboplatin, and fluorouracil could also be a second-line attempt to salvage MTX resistance instead of Act-D. Given the rarity of GTN and the relative efficacy and safety of Act-D, a clinical trial comparing second-line Act-D with other single-agent regimens is unlikely. Although the complete remission rates were significantly higher in etoposide-based regimens than in MTX-based and Act-D-based regimens in some studies [34], secondary malignancies associated with etoposide, especially leukemia, have been reported [34, 41]. According to toxicity data of included studies in this meta-analysis, no case of secondary malignancies has been observed in 987 and 687 patients treated with MTX and Act-D, respectively. Thus, second-line chemotherapy with Act-D among LRGTN has been regarded as a preferable attempt after MTX resistance. In order to reduce exposure to single-agent regimen with greater toxicity and combination chemotherapy, cancer centers should adopt more reasonable medication plan and management approach to deal with short-term and long-term side-effects, though the long-term toxicity of these drugs is difficulty to assess.

Some limitations of this meta-analysis should be stated. First, heterogeneity between the included studies was generally present in this meta-analysis, manifesting in the difference of drug dosages and cycles, criteria for defining complete response, pretreatment beta-hCG level, FIGO score, and follow-up time. However, subgroup analyses stratified by these factors were not possible because of unavailable information. Retrospective design of some studies was the inherent bias, while the results of subgroup analysis for complete remission were consistent with the pooled result that obtained from all studies. Second, the choice between a fixed-effect and random-effect model should not be solely based on a heterogeneity test, but one should choose the model fitting the sampling frame. When fixed-effect model was employed, it’s assumed that the true effect size of Act-D versus MTX is not differ from study to study. However, it’s probably not true unless all the studies are based on the same population. Conclusions drawn from pooled estimates using fixed-effect models are only true among the studies included in the meta-analyses, but would not be generalized beyond the population included in the analysis. Third, adverse events data were collected and graded according to different criteria, including CTCAE, WHO, and Gynecologic Oncology Group toxicity criteria. Few studies did not mention the methods or criteria for collecting adverse events, possibly depending on investigators’ evaluation or self-reporting by patients. Additionally, some treatment-related adverse events were not fully reported, we therefore could not make analyses for the toxicities. For some toxicities, sample sizes included in analyses are very small and indicates, therefore, a potential limitation when evaluating drug safety profile. Fourth, most of the studies have been performed by Asian and Latin America institutions, limiting the interpretation of the results for western populations. Fifth, the meta-analysis was based on summary data extracted from published articles and not on individual patient data. Finally, meta-analysis is inherently observational and it is possible that the results are affected by unmeasured cofounding factors.

## Conclusion

In this meta-analysis, clinical differences in efficacy and safety exist among Act-D and MTX for patients with LRGTN. We found that Act-D-based regimen has better efficacy profile in general, and MTX-based regimen was associated with less toxicities. These findings could optimize current treatment management and enhance future study design for LRGTN.

## Supplementary Information

**Additional file 1: Fig. S1**. Comparisons of ORs according to drug and study type (random-effects model).

**Additional file 2: Fig. S2**. Forest plots of pooled ORs for nausea (A) and vomiting (B) (random-effects model).

**Additional file 3: Fig. S3**. Forest plot of pooled OR for alopecia (random-effects model).

**Additional file 4: Fig. S4**. The horizontal box plots and funnel plots of ORs for complete remission.

**Additional file 5: Fig. S5**. The horizontal box plots and funnel plots of ORs for anaemia (A), leucopenia (B), neutropenia (C), and thrombocytopenia (D).

**Additional file 6: Fig. S6**. The horizontal box plots and funnel plots of ORs for constipation (A), diarrhea (B), nausea (C), and vomiting (D).

**Additional file 7: Fig. S7**. The horizontal box plots and funnel plots of ORs for alopecia (A), anorexia (B), fatigue (C), and liver toxicity.

**Additional file 8: Table S1**. Results of Begg’s adjusted rank correlation test and Egger’s test for adverse events.

## Data Availability

As this is a systematic review and meta-analysis, all eligible studies are listed in the reference list, and have been clearly listed in the manuscript. The datasets used and/or analysed during the current study are available from the corresponding authors on reasonable request.

## Abbreviations

GTN: Gestational trophoblastic neoplasia
WHO: World Health Organization
FIGO: International Federation of Gynecology and Obstetrics
LRGTN: Low-risk GTN
Act-D: Actinomycin-d
MTX: Methotrexate
5d-IV: Act-D the five-day Act-D regimen
MTX-FA: MTX-folinic acid regimen
RCTs: Randomized controlled trials
non-RCTs: Non-randomized studies
PRISMA: Preferred reporting items for systematic reviews and meta-analysis
ORs: Odds ratios
95%CIs: 95% confidence intervals
CTCAE: The Common Terminology Criteria for Adverse Events
GOG: Gynecologic Oncology Group
