# Supplementary figures and images for: Direct comparisons of efficacy and safety between actinomycin-D and methotrexate in women with low-risk gestational trophoblastic neoplasia: a meta-analysis of randomized and high-quality non-randomized studies
Source: BMC Cancer. 2021 Oct 18;21:1122. doi: 10.1186/s12885-021-08849-7 (PMC8524874; doi:10.1186/s12885-021-08849-7)

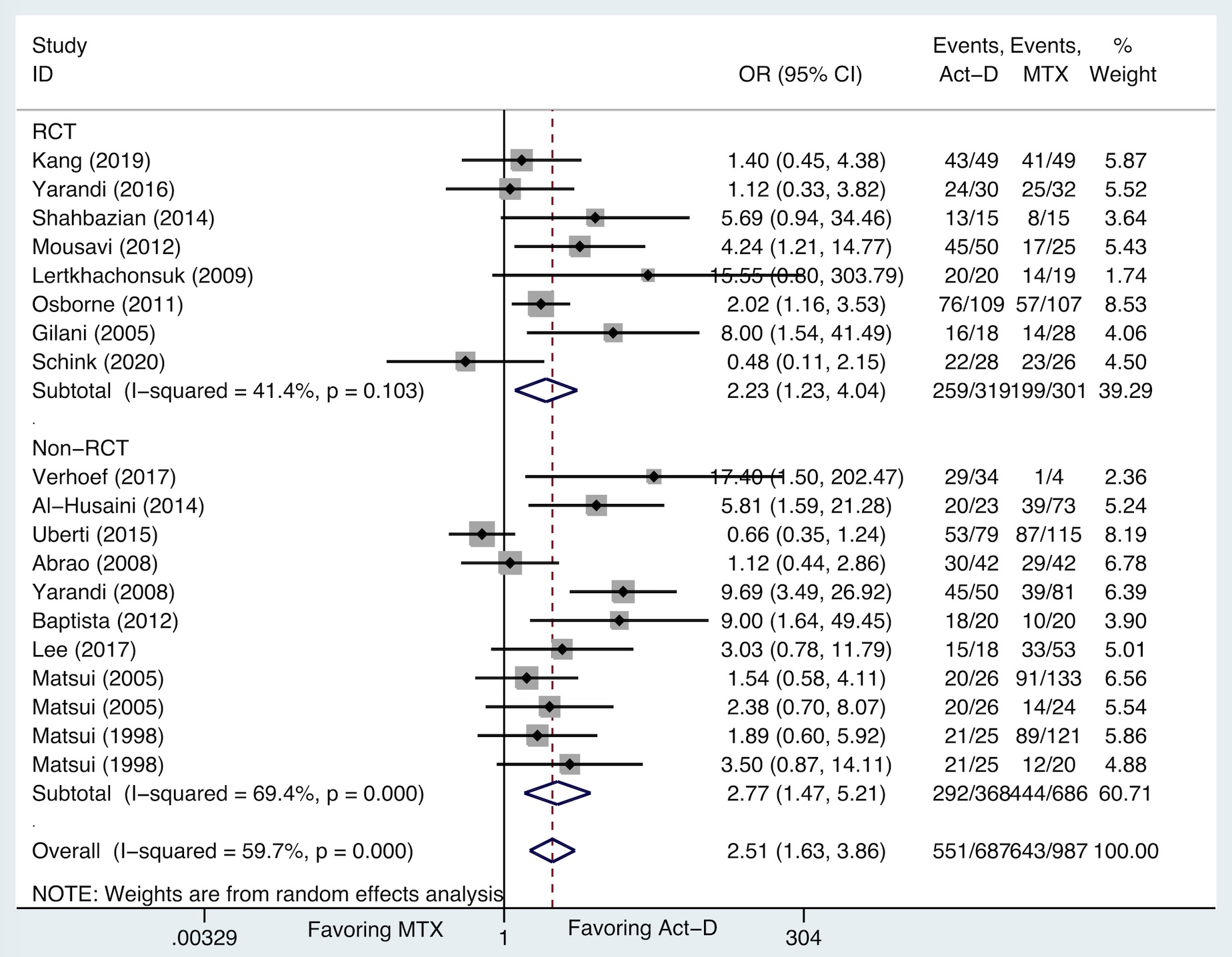

Supplement: Supplementary file 1 — Additional file 1: Fig. S1. Comparisons of ORs according to drug and study type (random-effects model). [file 12885_2021_8849_MOESM1_ESM.jpg]

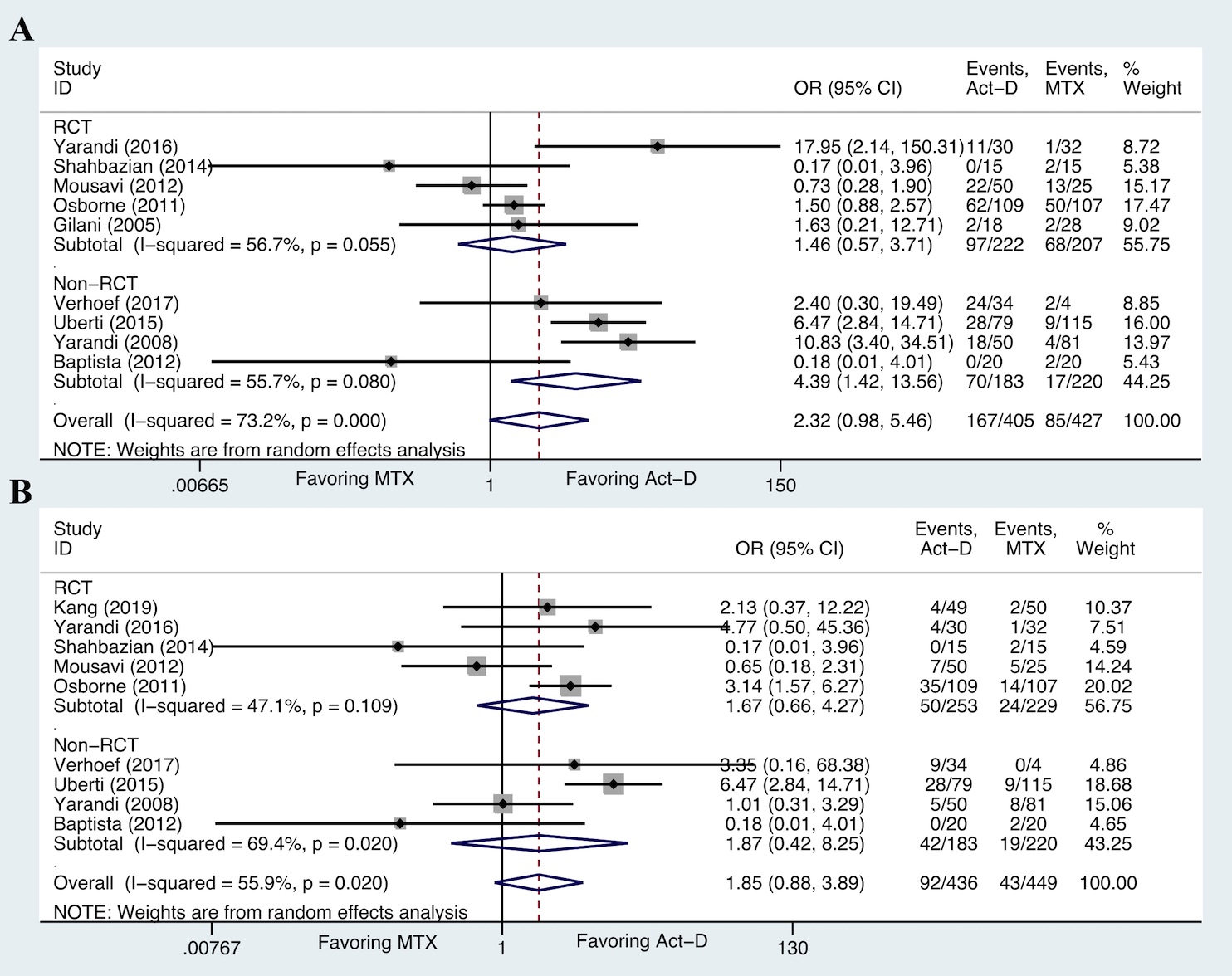

Supplement: Supplementary file 2 — Additional file 2: Fig. S2. Forest plots of pooled ORs for nausea (A) and vomiting (B) (random-effects model). [file 12885_2021_8849_MOESM2_ESM.jpg]

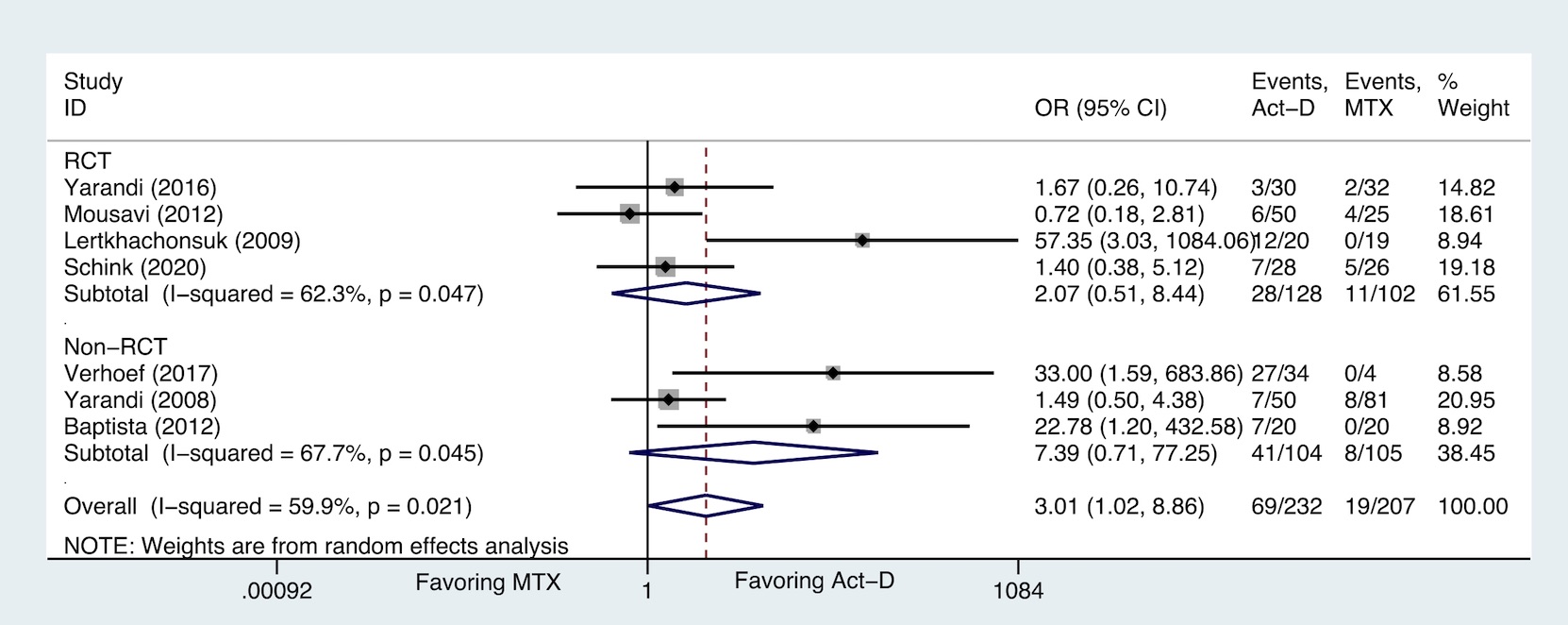

Supplement: Supplementary file 3 — Additional file 3: Fig. S3. Forest plot of pooled OR for alopecia (random-effects model). [file 12885_2021_8849_MOESM3_ESM.jpg]

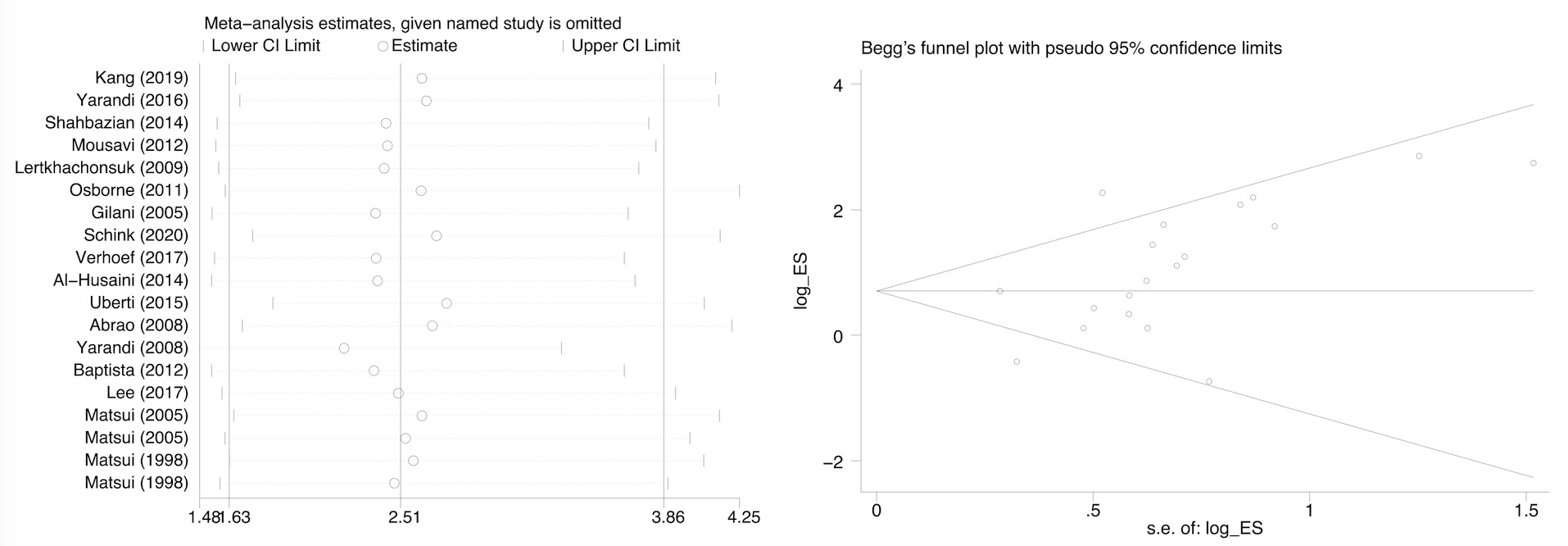

Supplement: Supplementary file 4 — Additional file 4: Fig. S4. The horizontal box plots and funnel plots of ORs for complete remission. [file 12885_2021_8849_MOESM4_ESM.jpg]

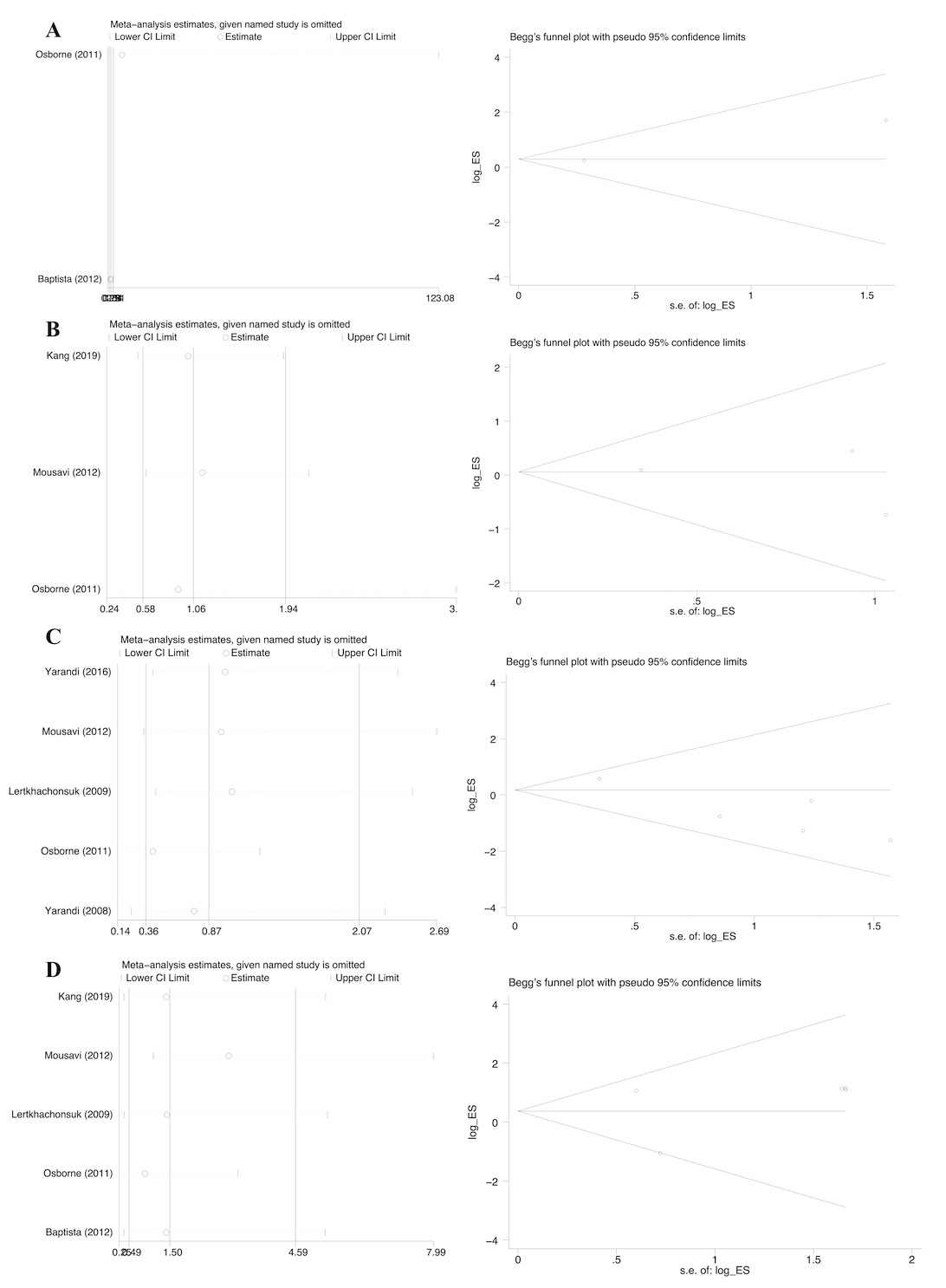

Supplement: Supplementary file 5 — Additional file 5: Fig. S5. The horizontal box plots and funnel plots of ORs for anaemia (A), leucopenia (B), neutropenia (C), and thrombocytopenia (D). [file 12885_2021_8849_MOESM5_ESM.jpg]

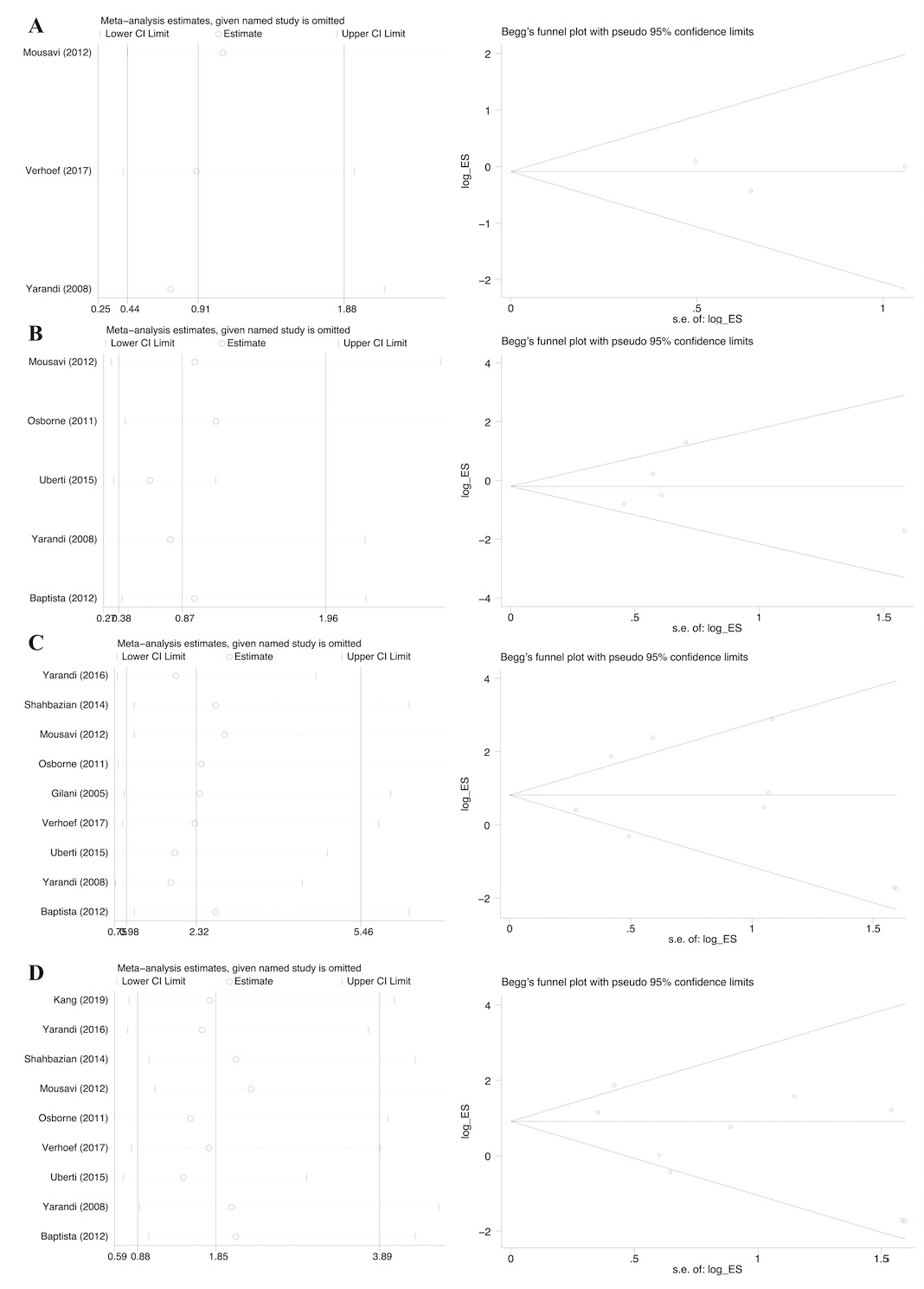

Supplement: Supplementary file 6 — Additional file 6: Fig. S6. The horizontal box plots and funnel plots of ORs for constipation (A), diarrhea (B), nausea (C), and vomiting (D). [file 12885_2021_8849_MOESM6_ESM.jpg]

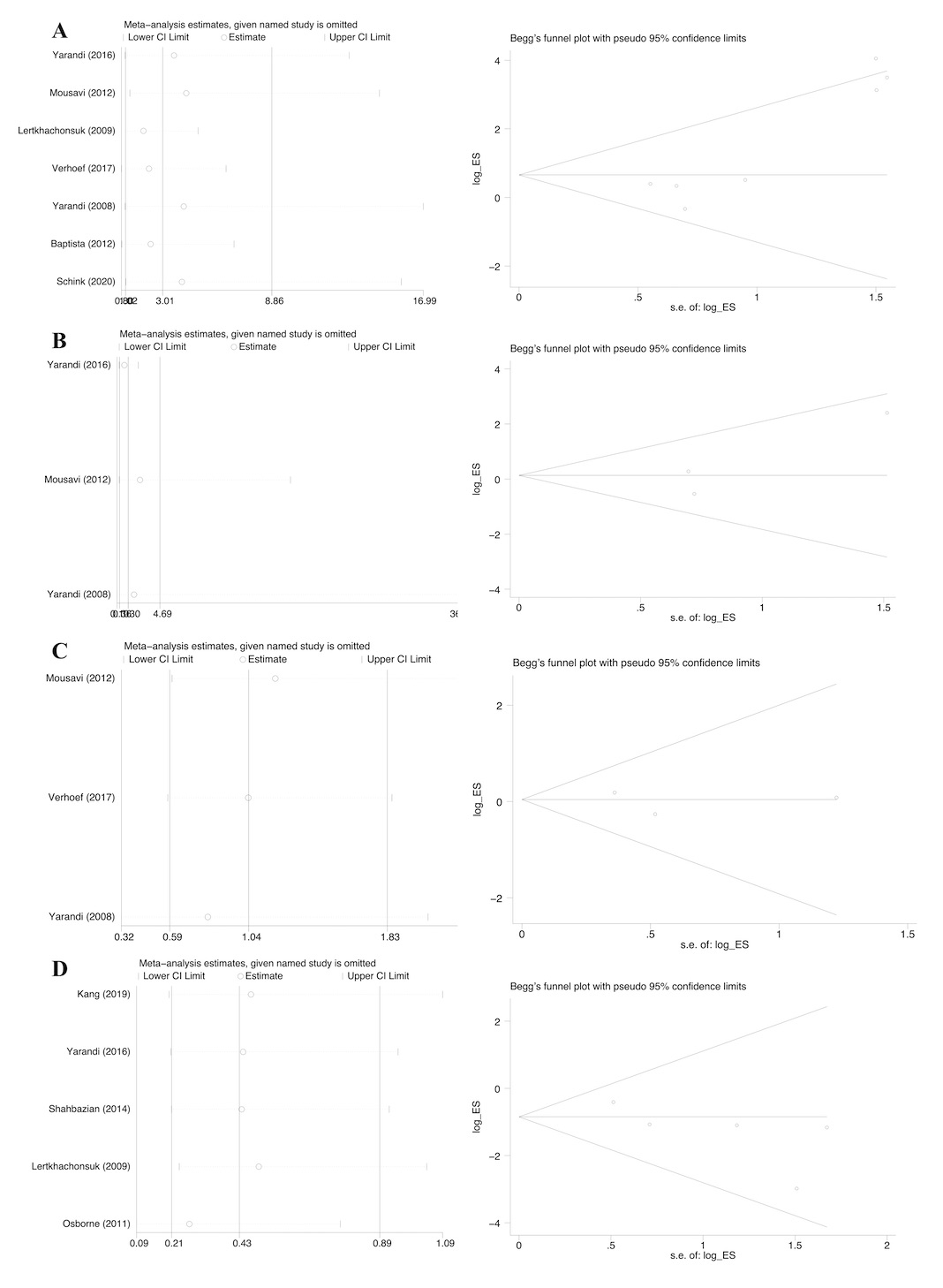

Supplement: Supplementary file 7 — Additional file 7: Fig. S7. The horizontal box plots and funnel plots of ORs for alopecia (A), anorexia (B), fatigue (C), and liver toxicity. [file 12885_2021_8849_MOESM7_ESM.jpg]
